# Role of senataxin in R-loop-mediated neurodegeneration

**DOI:** 10.1093/braincomms/fcae239

**Published:** 2024-07-15

**Authors:** Annapoorna Kannan, Shyni Gangadharan Leela, Dana Branzei, Laxman Gangwani

**Affiliations:** Center for Human Genetics, University of Oxford, Oxford OX3 7BN, UK; Bond Life Sciences Center, University of Missouri, Columbia, MO 65211, USA; Department of Veterinary Pathobiology, University of Missouri, Columbia, MO 65211, USA; The AIRC Institute of Molecular Oncology Foundation, IFOM ETS, Milan 20139, Italy; Istituto di Genetica Molecolare, Consiglio Nazionale delle Ricerche (IGM-CNR), Pavia 27100, Italy; Bond Life Sciences Center, University of Missouri, Columbia, MO 65211, USA; Department of Veterinary Pathobiology, University of Missouri, Columbia, MO 65211, USA

**Keywords:** R-loops, SETX, ZPR1, amyotrophic lateral sclerosis 4, spinal muscular atrophy

## Abstract

Senataxin is an RNA:DNA helicase that plays an important role in the resolution of RNA:DNA hybrids (R-loops) formed during transcription. R-loops are involved in the regulation of biological processes such as immunoglobulin class switching, gene expression and DNA repair. Excessive accumulation of R-loops results in DNA damage and loss of genomic integrity. Senataxin is critical for maintaining optimal levels of R-loops to prevent DNA damage and acts as a genome guardian. Within the nucleus, senataxin interacts with various RNA processing factors and DNA damage response and repair proteins. Senataxin interactors include survival motor neuron and zinc finger protein 1, with whom it co-localizes in sub-nuclear bodies. Despite its ubiquitous expression, mutations in senataxin specifically affect neurons and result in distinct neurodegenerative diseases such as amyotrophic lateral sclerosis type 4 and ataxia with oculomotor apraxia type 2, which are attributed to the gain-of-function and the loss-of-function mutations in senataxin, respectively. In addition, low levels of senataxin (loss-of-function) in spinal muscular atrophy result in the accumulation of R-loops causing DNA damage and motor neuron degeneration. Senataxin may play multiple functions in diverse cellular processes; however, its emerging role in R-loop resolution and maintenance of genomic integrity is gaining attention in the field of neurodegenerative diseases. In this review, we highlight the role of senataxin in R-loop resolution and its potential as a therapeutic target to treat neurodegenerative diseases.

## Teaser

Kannan *et al*. report that senataxin plays critical role in R-loop-mediated neurodegeneration associated with neurodegenerative disorders. Mutations in senataxin cause defects in R-loop homeostasis leading to neurodegeneration. *ZPR1* is a modifier of R-loop-mediated neurodegeneration caused by alteration of senataxin function in spinal muscular atrophy and amyotrophic lateral sclerosis 4.

## Graphical Abstract

**Graphical Abstract:**
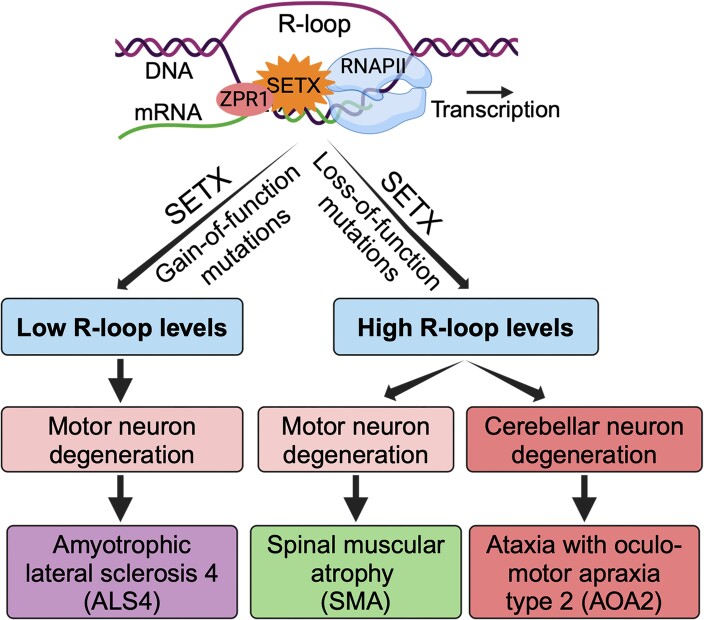

## Introduction

Several studies demonstrate that dysregulation of RNA metabolism by aberrant functioning of RNA-binding proteins (RBPs) plays a central role in disease pathogenesis, especially of neurodegenerative diseases. Defects in RBPs have a deleterious effect on neuron survival and functioning. Such defects are often associated with impaired RBP expression, cellular mislocalization of aggregated RBPs, dysregulation of various metabolic pathways by sequestering RNA and proteins and DNA damage caused by increased accumulation of R-loops affecting the genomic integrity. Senataxin (SETX) is one such RBP, known as a guardian of the genome, regulates diverse cellular processes linked to genomic integrity, including regulation of RNA metabolism, resolution of RNA:DNA hybrids of R-loops and DNA damage response (DDR) and repair. Interestingly, mutations in SETX with loss-of-function and gain-of-function are associated with distinct human neurodegenerative disorders. In this review, we shed light on the multifaceted role of SETX in cellular processes, its role in the pathogenesis of neurological disorders and its therapeutic implication as a potential modifier of disease phenotype.

## R-loops

R-loops are three stranded structures resulting during transcription in which the nascent RNA hybridizes to the template DNA strand forming initial RNA:DNA hybrids and displacing the non-template single-stranded DNA (ssDNA). As RNA/DNA hybrids elongate, they become R-loops. R-loops are relatively stable and account for 3–5% of the human genome.^1-3^ The stability of R-loops depends on the RNA structure, size and DNA sequence.^4^ The minimum length of 100 nucleotides of normal RNA is required to generate stable R-loops for quantitation. However, modification of uridines to 5-allylamine uridines (Uaa) requires only 50 nucleotides to form stable R-loops.^4^ The presence of G-quadruplex (G4) structures help stabilize the R-loops and are involved in regulatory processes, including DDX1 (RNA helicase)-dependent conversion of RNA G4 structures into R-loops during immunoglobulin H (IgH) class switch recombination^5^ and the CCCTC-binding factor (CTCF) binding, which plays a critical role in regulating chromatin architecture and gene expression.^6^

R-loops play important roles in several physiological cellular functions, which include class switch recombination of immunoglobulin G (IgG) in B cells, mitochondrial DNA replication, CRISPR-Cas9 gene edition, DNA repair and telomere homeostasis.^7-10^ R-loops are found frequently at GC-rich regions, high CpG islands containing promoters, transcription termination sites with rich GC skew or G4-containing sequences. R-loops preferentially accumulate at the promoter and termination regions of highly transcribed genes, implicating their potential as regulatory steps in transcription initiation and termination sites to regulate gene expression.^11-18^

Several factors prevent the formation of R-loops, including RNaseH, RNA/DNA helicases topoisomerase I (TOP I) and ribonucleoproteins (RNPs) involved in RNA metabolism. There are two types of RNaseH, RNaseH1 and RNaseH2, all of which can resolve RNA:DNA hybrids and R-loops.^19-21^ Regarding RNA/DNA helicases, SETX is the primary helicase that removes R-loops formed during transcription.^22-24^ Aquarius intron-binding spliceosomal factor,^25^ DEAD-box-like putative RNA/DNA helicase, DDX5,^26^ RECQL5,^27^ Pif1,^28^ BLM and Sgs1 (yeast orthologue of human BLM and RECQL5),^29,30^ DDX18,^31^ FANCM,^32,33^ Mph1 (yeast homologue of FANCM)^34^ and DHX9^35,36^ are other helicases involved in clearance of R-loops.^5,37^ The RNA helicase DDX19, a mRNA export factor, removes R-loops formed during replication stress.^38^ DDX23 is another RNA helicase that functions at the RNAPII pause site.^39^ TOP I and topoisomerase II (TOP II)^40-42^ relax the negative supercoiling of DNA formed behind elongating RNA polymerase II (RNAPII) and prevent R-loop formation. Moreover, topoisomerase, TOP3B, coordinates with the DEAD-box helicase DDX5 in resolution of R-loop resolution.^42,43^ Finally, various RNPs involved in RNA processing, splicing, packaging and export prevent R-loop formation by concomitantly binding to nascent RNA transcripts.^19-21,44^

### R-loop accumulation and neurodegenerative diseases

Accumulation of R-loops is associated with the pathogenesis of several neurodegenerative disorders. Such accumulation causes genomic instability due to DNA double-strand breaks (DSBs), hyper-mutation, hyper-recombination, transcription-associated recombination, gross chromosomal rearrangements, as reviewed in Aguilera and García-Muse,^7^ Crossley *et al.*^8^ Li and Manley,^45^ RNA processing defects^44,46^ and replication stress,^47^ as well as fragile site instability and chromosome loss if not properly cleared.^48^ Several neurological disorders, including ataxias, neuromuscular disorders and nucleotide repeat expansion disorders, result from mutation of genes involved in R-loop resolution.^14,15,49,50^ Specific examples of neurological disorders include nucleotide repeat expansion disorders such as Huntington’s disease and spinocerebellar ataxia type 1, which are caused by CAG repeats in *Huntingtin* and *ataxin 1* genes, respectively. Other neurological disorders included fragile X mental retardation or fragile X syndrome caused by CGG repeats in the *fragile X mental retardation 1* gene and Friedreich’s ataxia with GAA repeats in *frataxin* gene.^51,52^ R-loops formed following transcription of GC-rich trinucleotide repeats become highly stable due to the formation of G4 and triplex DNA structures in the non-transcribing DNA strand, which further stabilize the R-loops and increase the probability for DNA damage and genomic instability.^53-55^ R-loop accumulation is also reported in hexanucleotide GGGGCC repeat expansion in *C9ORF72* gene, which contributes to the molecular pathogenesis of amyotrophic lateral sclerosis with frontotemporal dementia (ALS-FTD).^56^ R-loop accumulation is reported in cells derived from patients with ataxia with oculomotor apraxia type 2 (AOA2) caused by mutations in the *SETX* gene.^57-59^ Recent studies have identified R-loop accumulation in neuromuscular disorders, also classified as motor neuron diseases. These include spinal muscular atrophy (SMA) caused by mutations in the *survival motor neuron 1* (*SMN1*) gene^60-62^ and amyotrophic lateral sclerosis 10 (ALS10) caused by mutations in the *TAR DNA binding protein* or *TDP-43* gene.^63^

## Functions of SETX

### R-loop resolution

The human *SETX* gene encodes an RNA/DNA helicase of 2677 amino acids, conserved across evolution, including the budding yeast orthologue known as *Splicing Endonuclease 1* (*SEN1*) gene that encodes similar RNA/DNA helicase.^22,64^ SETX is an ubiquitously expressed protein containing a protein–protein interaction domain in the N-terminal and a DEAD-box helicase domain, and nuclear localization signal in the C-terminal.^64,65^ SETX helicase domain is highly conserved and shows significant homology with helicase domains of Sen1,^66^ regulator of nonsense transcripts-1 and immunoglobulin mu DNA binding protein 2. SETX localizes in both the nucleus and the cytoplasm, suggesting cytoplasmic roles besides transcription-associated R-loop processing and metabolism.^67,68^ The growing SETX protein interactome implicates a broader role of SETX in the function of RNA processing, including transcriptional termination and maintenance of genomic stability.^23,69-71^

Significant insight into the function of SETX is gained from studies of budding yeast Sen1. Sen1 is a highly conserved RNA and DNA helicase that belongs to the superfamily 1B of helicases.^72,73^ Sen1 interacts with RNA-Pol I, RNA-Pol II and RNA-Pol III.^74,75^ Sen1 is important for transcription termination of genes transcribed by three RNA polymerases and contributes to the processing of diverse RNAs, including tRNA, rRNA, snRNA and snoRNA.^23,76-84^ Sen1 interacts with multiple proteins involved in RNA synthesis, processing, transcription termination, RNP assembly and maturation, as well as DNA repair.^85,86^ Similarly, human SETX regulates gene expression by controlling transcription initiation, termination and pre-mRNA splicing.^87^ SETX also interacts with several proteins involved in transcription, splicing, RNA processing and stability factors, R-loop resolution and DNA repair, including nucleolin, RNAPII, SMN, hnRNPs, poly(A)-binding proteins 1 and 2, SF3B1 and SAP155,^69^ ZPR1^88^ and BRCA1.^89^

The functions of SETX and Sen1 in R-loop processing and transcription termination demonstrate the conservation of R-loop metabolism among eukaryotes.^24,73,90,91^ Both Sen1 and SETX, through their interactions with RNA polymerases, are targeted to chromatin to continuously surveil the local chromatin landscape for the presence of R-loops. When encountered, they mediate timely removal of R-loops by unwinding the nascent RNA from RNA:DNA hybrids through the highly conserved DNA/RNA helicase activity at transcriptionally active sites and stalled elongation complexes.^72,85,91-93^ Mutations in the catalytic domain of Sen1 confer defective transcription termination and increased transcriptional readthrough of several transcription units on small coding and non-coding genes, rDNA and tRNA. These events cause reduced gene expression, accumulation of R-loops and increased recombination.^19,22^ Moreover, mutations in Sen1 also cause increased transcription associated with genome instability.^22,94^ In summary, the resolution of R-loops by SETX prevents DNA damage and chromosomal translocations and helps maintain the integrity of the genome.^87,90^

### Transcription termination

Transcription termination of protein coding genes involves cleavage at the polyadenylation [poly(A)] site by the cleavage and polyadenylation factor-cleavage factor IA and IB (CPF-CF1A and CF1B) complex of the pre-mRNA transcript and addition of a poly(A) tail.^95,96^ Yeast Sen1 is involved in poly(A)-dependent termination.^23,69,97,98^ The C-terminal domain of Sen1 has a sequence for nuclear localization and interacts with Glc7p, a protein phosphatase subunit of the cleavage/polyadenylation factor.^74,99,100^ Sen1 interacts with RBPs, Nab3 and Nrd1 to form the Nrd1–Nab3–Sen1 (NNS) complex. NNS complex comprises two RBPs, Nrd1 and Nab3, in addition to Sen1 helicase,^99,101^ which carries out the Sen1-mediated transcription termination of non-coding RNAs (ncRNAs) in yeast. ncRNAs are polyadenylated by the Trf4, a subunit of the TRAMP complex mediating this process. Nrd1 of the NNS complex interacts with Trf4 of the TRAMP complex to promote the 3′-end trimming of ncRNAs and degradation of unstable RNAs in the nuclear exosome bearing the Rrp6 exonuclease.^83,101-105^ The NNS complex is also required for the biogenesis of most of the snRNA and snoRNAs and their 3′-end maturation by the exosome.^79^ Unlike the poly(A) pathway, the NNS pathway is not conserved in higher eukaryotes. Sen1 induces transcription termination in both cases by unwinding the RNA:DNA hybrid formed within the transcription bubble.^81,86,106^

In eukaryotic RNAPII, the conserved C-terminal arginine residue R1810 undergoes symmetrical dimethylation (me2 s) by protein arginine methyltransferase 5. Methylation of arginine helps to recruit the SMN protein and causes the formation of a ternary complex SETX–RNAPII–SMN, required to resolve RNA:DNA hybrids in the transcription termination regions. Mutation in the RNAPII R1810 residue results in the disruption of RNAPII and SMN binding, causing the accumulation of R-loops in the transcription termination regions.^77^

### Resolution of transcription and replication collision conflicts

SETX also has a role in DDR by co-localizing at transcriptionally induced DNA damage sites. SETX forms nuclear foci during the S-phase of the cell cycle. These foci represent sites of DNA polymerase/RNAPII collision and co-localize with DDR markers 53BP1 and γH2AX. SETX undergoes SUMO-2 modification specifically during the early S-phase, facilitating SETX accumulation into these foci.^23,66,75^ At these nuclear foci, RNAPII collides with the replication fork and results in the halt of RNA elongation. SETX utilizes its helicase activity to unwind the R-loop formed behind the stalled RNAPII. SETX then directs incomplete RNA transcripts to the nuclear exosome for degradation through interaction with components of the nuclear exosome complex, Rrp45 and exosome component 9 (Exosc9), which require SETX SUMOylation at the N-terminus.^107,108^ The exosome complex is the major eukaryotic 3′ → 5′ exonuclease, conferring accurate degradation of RNAs in the nucleus and cytoplasm and playing a critical role in RNA turnover and quality control.^109^ The SUMOylation of SETX and the interaction of SUMOylated SETX with Rrp45 and Exosc9 are disrupted in AOA2 but not in amyotrophic lateral sclerosis 4 (ALS4). The loss of SETX, Rrp45 and Exosc9 interaction in AOA2 cells may contribute to a defective DDR and repair of DSBs, commonly present in these cells.^110^

Sen1 also binds to the replisome complex by interacting with Ctf4 and Mrc1 components of the replication fork through its N-terminal domain. Association of Sen1 to the replisome is abolished upon deletion of *CTF4* or *MRC1* genes.^111^ The association of Sen1 with the replication fork complex is critical for the timely removal of R-loops promoting fork progression across RNAPII transcribed genes. Altogether, these results highlight a key functional role of Sen1 in replication fork progression during DNA replication and chromosome stability.^94,111^ A recent study also shows that Sen1 acts as a key regulator in resolving transcription-driven conflicts.^112^

### Telomere stability

Sub-telomeric and telomeric DNAs are transcribed by RNAPII into telomere repeat containing ncRNA (TERRA/TelRNA).^113,114^ Transcription-dependent binding of SETX to telomeric and sub-telomeric DNA and its inhibition by amanitin suggests a possible involvement of SETX in the regulation of TERRA expression.^115^ AOA2 patient cells possessing constitutively shortened telomeres show increased sensitivity with further shortening of telomere length when treated with camptothecin and X-rays, suggesting a possible involvement of SETX in maintaining the telomere length and stability via a mechanism tied to TERRA formation.^113-115^

### Transcription-coupled DNA damage and repair

In addition to RNA metabolism, SETX plays a crucial role in DNA integrity and genome stability. SETX associates with many DDR and repair proteins, including γH2AX, 53BP1, MDC1, XPA, FANCD2, ATRIP, BRCA1, DNA-PKCs, MRE11, RAD50 and PIAS1, involved in the repair of DNA lesions caused by oxidative DNA damage, DNA single-strand breaks (SSBs) and DNA DSBs. Loss of SETX causes increased accumulation of DNA damage,^59,75,89,108,116^ potentially due to defective DNA repair. The N-terminal domain of Sen1 interacts with nucleotide excision repair factor, Rad2, a single-strand DNA endonuclease required for transcription-coupled DNA repair.^74,117-119^

SETX is also recruited to DSBs induced in transcriptionally active loci where it unwinds the induced RNA:DNA hybrids. At transcriptionally active loci, DSBs induce RNAPII stalling, thereby stabilizing the R-loop formation. Genome-wide studies revealed R-loop accumulation on regions flanking DSBs, with these levels reducing upon SETX binding.^45,120,121^ At DSB sites, SETX increases RAD51 recruitment and reduces illegitimate resection of broken ends, thereby preventing translocation and promoting cell viability following DSB production in transcriptionally active genes. Thus, SETX loss contributes to neuronal loss in AOA2 and ALS4 pathology.^90^ SETX-associated nuclease 1 (SAN1) interacts with SETX. SETX recruits SAN1 to sites of inter-strand DNA cross-links (ICLs) and together they mediate their repair. The cascade of events here is triggered by the stalling of the transcription complex and subsequent formation of R-loops at ICL lesions. SAN1 repairs ICLs independently of the Fanconi anaemia pathway, thereby acting as a parallel pathway to protect cells from ICL lesions. SAN1–SETX interaction is crucial for SAN1-mediated repair of ICLs.^122^

SETX's DNA repair involvement also relies on its direct interaction with BRCA1, a key player in DSB repair by homologous recombination (HR). Genome-wide studies suggested that BRCA1 binds to R-loops formed at transcription termination regions of active gene loci.^89,123^ It is proposed that BRCA1 mediates the recruitment of SETX and suppresses co-transcriptional R-loop-mediated DNA damage. Disruption of BRCA1–SETX interaction causes R-loop-induced SSBs in non-template DNA strand that are marked by the accumulation of γH2AX foci, suggesting a function of SETX–BRCA1 complexes in DNA repair. Sen1 has also been shown to play a direct role in transcription-coupled DNA repair.^124^

## SETX mutations and neurodegenerative disorders

**Figure 1:**
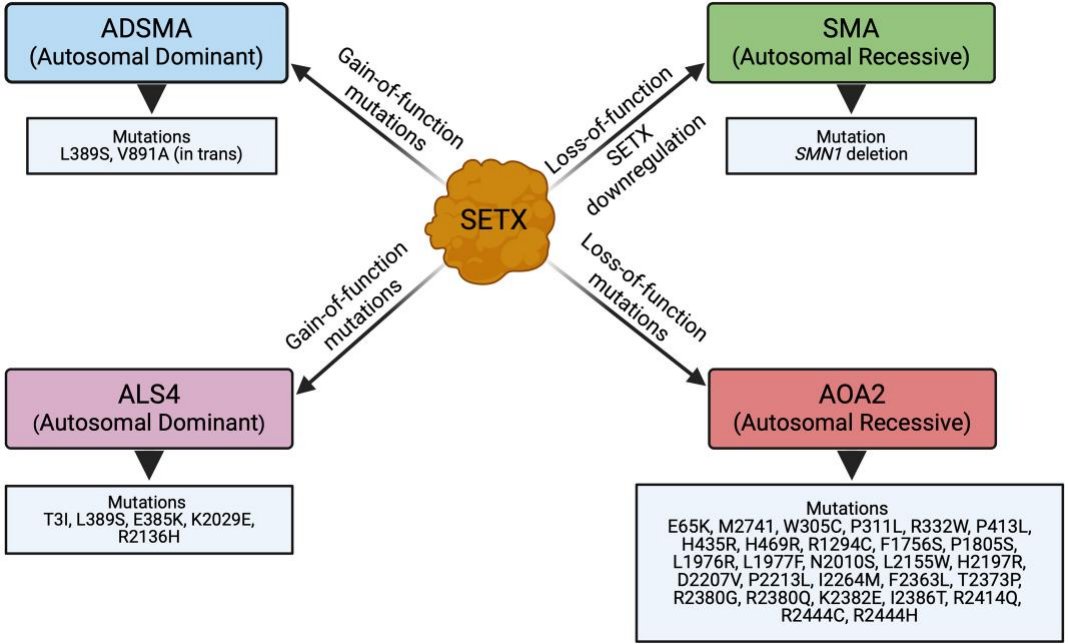
**Mutations in SETX that result in gain of function, loss of function or SETX downregulation cause distinct human genetic neurodegenerative disorders.** Graphical illustration of neurodegenerative disorders caused by SETX mutations with gain of function, loss of function and SETX downregulation. Gain-of-function mutations cause autosomal dominant ALS4 and autosomal dominant SMA (ADSMA); loss-of-function mutations cause autosomal recessive AOA2; and the downregulation of SETX levels associated with autosomal recessive SMA.

The role of SETX in neurodegeneration was suggested after the identification of SETX mutations in patients with neurodegenerative disorders.^66^ Mutations in the *SETX* gene are associated with different neurodegenerative disorders such as autosomal dominant ALS4,^65,125-129^ autosomal recessive AOA2^67,130-133^ and autosomal dominant SMA (Fig. 1).^134^ A broad-spectrum of homozygous mutations in SETX has been identified, of which most of the missense mutations are clustered around the conserved helicase domain or the amino terminal domain.^66^ Such clustered mutations in SETX cause loss of function resulting in AOA2, characterized by cerebellar ataxia with occasional oculomotor apraxia, cerebellar atrophy and peripheral neuropathy.^64,130,131^ Analysis of cells derived from AOA2 patients suggests that loss of SETX function results in R-loop accumulation.^59^ Studies with iPSCs and neural progenitors derived from AOA2 patient cell lines reveal increased levels of R-loop accumulation and increased sensitivity to DNA damaging agents such as H_2_O_2_, camptothecin and mitomycin C as well as high susceptibility to oxidative DNA damage-induced cell death. The abnormal expression of SOD1 in patient-derived AOA2 cells may be a cause of increased levels of H_2_O_2_, resulting in oxidative DNA damage.^59,110,115,116^ A recent study provided insight into Sen1 structural changes responsible for the loss of SETX helicase activity and defects in RNA-binding ability of SETX mutations associated with AOA2.^73^ The role of SETX in ALS4 pathogenesis has been recently reviewed.^135^

The decrease in SETX levels results in the accumulation of R-loops in cells derived from SMA patients.^61^ The knockdown of SETX in mammalian cells results in accumulation of R-loops, suggesting that loss of function or decrease in SETX levels causes a disease-related phenotype of R-loop accumulation.^60^ SMA is caused by homozygous mutations/deletions of the *SMN1* gene resulting in low levels of the ubiquitously expressed SMN protein.^136,137^ SMA is characterized by the progressive degeneration of motor neurons in the anterior horns of the spinal cord results in muscle weakness, respiratory failure and death.^138^ Depletion of SMN results in reduced formation of snRNPs resulting in widespread splicing defects. Global splicing defects caused by chronic low levels of SMN in SMA may be a cause of splicing inefficiency of SETX pre-mRNA resulting in reduced full-length SETX transcripts, thus explaining the low levels of the SETX protein.^139-142^ A decrease in SETX levels causes accumulation of R-loops and DNA damage in SMA patient fibroblasts and spinal cord motor neurons derived from SMA mice.^61^

**Figure 2:**
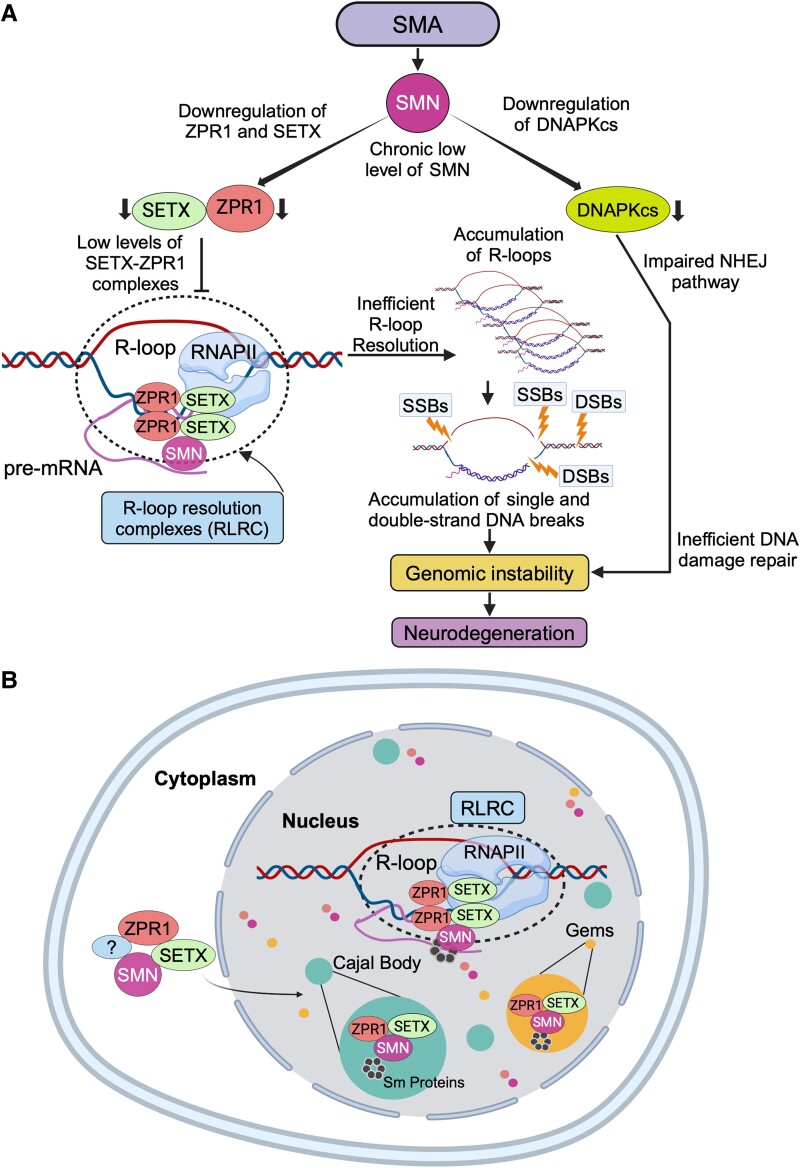
**Molecular mechanism of SETX-dependent and R-loop-mediated neurodegeneration associated with SMA pathogenesis.** (**A**) Chronic low levels of SMN cause downregulation of ZPR1 and SETX in SMA. The low endogenous levels of SETX–ZPR1 complexes result in poor assembly of RLRCs onto RNA:DNA hybrids during transcription, resulting in accumulation of R-loops. Increased accumulation of R-loops triggers SSBs and DSBs and causes activation of DDR pathways in SMA. DSB repair in neurons is predominantly mediated by NHEJ, which relies on the activation of DNA-PKcs. Chronic SMN deficiency also causes marked decrease in DNA-PKcs levels and its activation, resulting in inefficient NHEJ-mediated DNA repair in SMN-deficient neurons. Inefficient DSB repair in neurons results in accumulation of DNA damage leading to genomic instability and degeneration of neurons. (**B**) Cellular localization of SETX with core component of RNA resolution complexes (RLRC), including ZPR1, RNAPII and RNA:DNA hybrids. SETX is present in the cytoplasm and in the nucleus. SETX is an ATP-dependent RNA/DNA helicase that unwinds RNA:DNA hybrids formed during transcription. SETX interacts with ZPR1, RNAPII and RNA:DNA hybrids and co-localizes in sub-nuclear bodies, gems (SMN containing sub-nuclear bodies) and Cajal bodies. SETX is recruited onto R-loops by ZPR1. SETX, ZPR1 and RNAPII form endogenous complexes that assemble *in vivo* onto RNA:DNA hybrids formed during transcription and referred as to RLRC.

Investigation of the molecular mechanism of motor neuron degeneration provided insight into the role of SETX in R-loop-mediated neurodegeneration in SMA. Chronic low levels of SETX in SMA resulted in a higher ∼8-fold accumulation of R-loops in neurons compared to ∼2-fold in fibroblasts indicating a greater amount of DNA damage accumulation in neurons than in fibroblasts and suggesting a higher efficiency of DNA repair is required to prevent genomic instability and neurodegeneration.^61^ Because the motor neurons degenerate in SMA, these findings suggested a likely defect in the DNA repair mechanisms. DNA damage, specifically DSBs, is repaired by two main pathways in dividing cells (fibroblasts): HR and non-homologous end joining (NHEJ), with the latter further divided into canonical NHEJ (c-NHEJ) and alternative NHEJ (alt-NHEJ).^143^ However, neurons rely on NHEJ for DSB repair.^144^ Investigation of the activation of NHEJ in SMA neurons demonstrated that DNA-activated protein kinase catalytic subunit (DNA-PKcs), which is critical for this branch, is downregulated in SMA patient spinal cords and motor neurons from SMA mice (Fig. 2A).^61^ Accumulation of SETX-dependent R-loops and DNA damage combined with inefficient NHEJ-mediated DSB repair due to DNA-PKcs deficiency leads to genomic instability and degeneration of motor neurons in SMA.^60,61,145^

ALS4 is a juvenile form of autosomal dominant neuromuscular disease characterized by progressive degeneration of upper and lower motor neurons in the brain and spinal cord and results in progressive muscle weakness, muscle wasting, atrophy and spasticity.^128^ In contrast to the SETX mutations in AOA2 that cause loss of function, SETX mutations associated with ALS4 result in gain of function in SETX R-loop resolution activity leading to neurodegeneration.^65,125,146,147^ An increase in SETX-dependent R-loop resolution activity results in fewer R-loops in ALS4 patient cells. Low levels of R-loops in promoters allow modification of regulatory regions such as DNA methylation that regulate transcription.^12,13,148^ In ALS4 cells, reduced R-loops allow DNA methylation, which decreases the expression of BAMBI, a negative regulator of the transforming growth factor beta (TGF-β) pathway, therefore causing the activation of the TGF-β signalling that may mediate neurodegeneration.^149^ Alteration of the TGF-β signalling pathway is just one example, but there may be several other genes or pathways altered in response to the low levels of R-loops that contribute to ALS4 pathogenesis. Thus, further investigation of the effects of reduced R-loops in neurons will provide better insight into the cause of neurodegeneration in ALS4.

## Mechanism of SETX-dependent R-loop resolution

Understanding the molecular mechanism of R-loop resolution will provide critical insight into modulating levels of R-loops and managing pathological conditions. The molecular mechanism of R-loop resolution is unclear. However, some progress has been made towards identifying critical steps involved in SETX-mediated R-loop resolution under normal and ALS4 disease conditions.^88,150^

The helicase activity of SETX is involved in the unwinding of RNA:DNA hybrids,^24^ one of the initial steps in the resolution of R-loops. SETX interacts with RNAPII,^69^ but how the helicase activity of SETX is regulated and how the protein is recruited to R-loops are still unclear. A recent study showed that the zinc finger protein 1 (ZPR1) binds to SETX and is required to recruit SETX onto R-loops. Moreover, ZPR1 may regulate the helicase activity of SETX.^88^ ZPR1 is an essential protein that is evolutionarily conserved in eukaryotes.^151-153^ ZPR1 is shown to be involved in the regulation of transcription, cell growth and cell cycle progression.^154,155^ ZPR1 also interacts with RNAPII and is a part of transcription complexes.^62^ In addition, ZPR1 interacts with several proteins including the EGF receptor, eukaryotic translation elongation factor 1A and SMN.^152,154,156^ Notably, the interaction of ZPR1 with SMN is disrupted in SMA patients that have mutations in the *SMN1* gene.^156^ ZPR1 is required for the accumulation of SMN in sub-nuclear bodies, including gems (SMN containing nuclear bodies) and Cajal bodies (Fig. 2B). The disruption of ZPR1–SMN complexes causes mislocalization of SMN in SMA patient cells and motor neurons. ZPR1 plays a critical role in neuron survival. ZPR1 deficiency causes degeneration of spinal cord motor neurons and cerebellar granule neurons and results in the degeneration of nerves in the peripheral nervous system.^145,157-159^ Although ZPR1 is essential for neuronal cell viability, the essentiality mechanism was unclear until recently. Recent studies have provided insight into the function of ZPR1 in the resolution of R-loops, which may likely be its essential cellular function because impaired R-loop resolution would result in cell death. ZPR1 deficiency causes accumulation of R-loops and DNA damage in dividing cells and neurons.^62,88^

**Figure 3:**
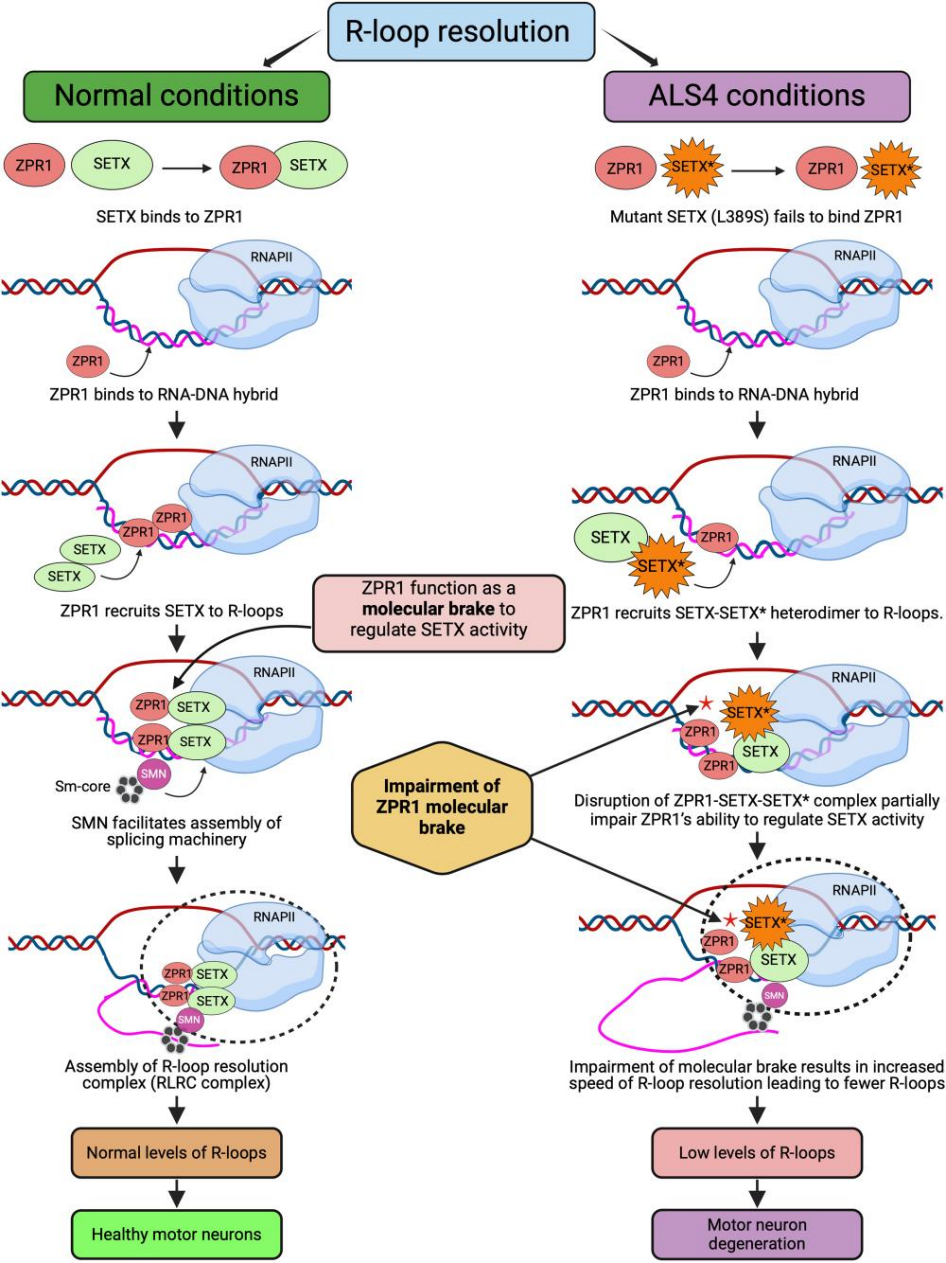
**The molecular mechanism of R-loop resolution in normal and disease conditions.** Left panel: Mechanism of R-loop resolution under normal conditions in mammalian cells. The key steps that contribute to R-loop resolution in mammalian cells are as follows: (i) ZPR1 binds to RNA/DNA hybrids, (ii) ZPR1 recruits SETX onto R-loops to initiate *in vivo* assembly of the core RLRC during transcription and (iii) SMN-dependent splicing machinery recruited to RLRC to initiate pre-mRNA processing. ZPR1 regulates the activity of SETX by controlling the speed of SETX-dependent R-loop resolution. ZPR1 regulates the R-loop resolution activity of SETX and thus may function as a ‘molecular brake’ to modulate the speed of SETX-dependent R-loop resolution and contributes to the survival and maintenance of neurons. Right panel: Mechanism of R-loop resolution under disease conditions in ALS4 patient-derived fibroblasts. Under ALS4 disease conditions, the following key steps contribute to R-loop resolution and disease pathogenesis: (i) interaction of ZPR1 with mutant SETX (L389S) shown as SETX* is disrupted. ZPR1 fails to recruit mutant SETX homodimer (SETX*–SETX*) but recruits heterodimer (SETX–SETX*) to R-loops. However, partial disruption of molecular interaction between ZPR1 and SETX* in the ZPR1–SETX–SETX* complex impairs ZPR1's ability to regulate R-loop resolution activity of mutant SETX* resulting in fewer R-loops in ALS4. In summary, mutations in SETX disrupt its interaction with ZPR1 causing partial impairment of the molecular brake, resulting in increased speed (activity) of SETX-dependent R-loop resolution (gain of function). The low levels of R-loops alter gene expression resulting in dysregulation of the TGF-β signalling pathway critical for neuron survival.

The core components of R-loop resolution complexes (RLRCs) bind directly to RNA:DNA hybrids. Other components critical for R-loop resolution, such as SMN, can directly bind to RNAPII, a core component of RLRC, and contribute to R-loop resolution. ZPR1 and SETX can independently bind to RNAPII and RNA:DNA hybrids. SETX interacts with ZPR1, co-localizes with ZPR1 in sub-nuclear bodies and forms *in vivo* complexes with R-loops. ZPR1 recruits SETX to R-loops and is critical for *in vivo* assembly of the RLRC comprising SETX–ZPR1–RNA:DNA–RNAPII.^88^ Of interest, the interaction of SETX with ZPR1 is disrupted in fibroblasts derived from ALS4 patients with SETX (L389S) mutation. Mutation in SETX causes mislocalization of SETX, which fails to accumulate in sub-nuclear bodies and is redistributed in the nucleoplasm. However, the levels of ZPR1 and SETX are not altered in ALS4.^88^ Disruption of ZPR1 interaction with SETX results in reduced recruitment of SETX onto R-loops.^88^ Partial recruitment of SETX by ZPR1 onto R-loops is due to two reasons: (i) ALS4 is an autosomal dominant disease caused by heterozygous SETX mutation, and (ii) SETX forms a dimer, with its dimerization ability not affected by the L389S mutation, suggesting that the SETX can form homo and heterodimers.^71^ Thus, ZPR1 may interact and recruit SETX–SETX (wild-type) homodimer and SETX–SETX* (wild-type mutant) heterodimer but fails to interact or recruit SETX*–SETX* (mutant–mutant) homodimer.^88^ One of the important questions addressed by a recent study is how mutation in SETX results in gain of function leading to fewer R-loops in ALS4. The study demonstrated that ZPR1 interacts with SETX and may function as a ‘molecular brake’ to regulate the speed of SETX-dependent R-loop resolution activity (Fig. 3). Therefore, suboptimal ZPR1 interaction with SETX may cause failure of this molecular brake, resulting in increased speed of SETX-dependent resolution of R-loops that leads to fewer R-loops in ALS4.^88^

## Role of SETX as a potential modifier of neurodegenerative diseases

The dysregulation of R-loop metabolism as a common pathogenic mechanism in mediating neurodegeneration in neurodegenerative disorders with either increased levels of R-loop accumulation, such as in SMA and AOA2, or with reduced levels of R-loops, such as in ALS4, suggests that modulation of R-loop levels could be exploited as a potential therapeutic method. The emerging connections between R-loop levels and proteins with the potential to regulate R-loop resolution, such as SETX, ZPR1 and other components of RLRC, could be potential modifiers of neurodegenerative diseases. Initially, ZPR1 was identified as a modifier of SMA disease. However, the precise mechanism of rescue of the disease phenotype was unclear.^156-161^ Recent studies demonstrated that SETX and SMN are downstream targets of ZPR1. Overexpression of ZPR1 in SMA patient cells, motor neurons and SMA mice resulted in upregulation of SMN and SETX levels that decreased pathogenic R-loop levels, reduced DNA damage and motor neuron degeneration and improved the lifespan of SMA mice.^61,62^ Further, overexpression of SETX in SMA patient cells and motor neurons reduced R-loop levels and DNA damage and prevented degeneration of spinal cord motor neurons.^62^ Together, these findings demonstrated that the R-loop-mediated toxic effects are rescued by ZPR1 and SETX, establishing their role as potential modifiers of disease severity by their ability to modulate the levels of pathogenic R-loops in SMA.^60-62,88,150^

Amyotrophic lateral sclerosis 4, caused by SETX mutation, is a unique and very interesting case of neurodegenerative disease characterized by reduced levels of R-loops. More interesting and important aspects of regulating R-loop levels emerged from the recent findings that ZPR1 can regulate the activity of SETX-mediated R-loop resolution in ALS4 patient cells. ZPR1 overexpression resulted in an increase of R-loop levels in normal and ALS4 cells. ZPR1 increased the levels of SETX that elevated the recruitment of SETX onto R-loops and the formation of ZPR1–SETX complexes, which allowed ZPR1 to control SETX-dependent R-loop resolution activity and restore normal levels of R-loops in ALS4 patient cells.^88^ In addition, cell models harbouring the *C9ORF72* sense repeats also show accumulation of R-loops and DSBs, which can be rescued by SETX overexpression, suggesting a potential therapeutic role of SETX in overcoming R-loop-associated defects and DNA damage in nucleotide repeat-associated neurodegenerative disorders.^162^ A recent study showed that SETX is a significant modifier of C9ORF72-related ALS-FTD disease severity caused by G4C2 and arginine containing dipeptide repeat toxicity.^163^

Together, these findings opened the door to exploring the therapeutic potential of the SETX–ZPR1 axis in regulating pathogenic R-loops in neurodegenerative diseases subject to abnormal levels of R-loops such as SMA and ALS4. It is possible that future research in understanding pathological mechanisms of neurodegenerative disorders will reveal others with defects in R-loop resolution, including AOA2, ALS-FTD, ALS10, Huntington’s disease, spinocerebellar ataxia, fragile X mental retardation or fragile X syndrome and Friedreich’s ataxia. The role of SETX may also be examined in other cellular processes correlated with neurological disorders, such as autophagy and mitochondrial dysfunction that are associated with the pathogenesis of Alzheimer's disease and Parkinson's disease. SETX regulates the expression of different autophagy genes, and differential expression of SETX has an adverse effect on the autophagic process.^164^ Defective autophagy is reported in AOA2 and ALS4 patient cells.^165^ Mutant SETX also shows defective redox signalling and severe loss of mtDNA with increased ROS production in ALS-FTD caused by the *C9ORF72* mutation, implicating the critical functional role of SETX in regulating redox homeostasis.^166,167^ Notably, R-loop accumulation at microRNAs (miRNAs) plays a role in the co-transcriptional processing of primary miRNAs.^168^ Of interest, there is convincing evidence of involvement of miRNAs and mitochondrial miRNAs in Alzheimer's disease pathogenesis; however, the role of R-loops in altering miRNAs in the context of Alzheimer's disease remains to be examined.^169-172^ Further, reports of DNA damage and defects in DNA repair associated with reduced DNA-PKcs and the possibility of R-loops in mediating transcriptional alterations in Alzheimer's disease related gene expression and DNA damage call for further investigations.^173-176^ In conclusion, SETX, a core component of the R-loop resolution machinery that functions in collaboration with other critical factors, such as ZPR1, may be a potential therapeutic target for a broad range of neurodegenerative diseases.

## Data Availability

Data sharing is not applicable to this article as no new data were created or analysed in this study.
